# FcRγIIA attenuates pathology of cutaneous leishmaniasis and modulates ITAMa/i balance

**DOI:** 10.1186/s13071-024-06593-y

**Published:** 2024-12-18

**Authors:** Ikram Hammi, Julien Giron-Michel, Myriam Riyad, Khadija Akarid, Damien Arnoult

**Affiliations:** 1 Health & Environment Laboratory, Ain Chock Faculty of Sciences, Hassan II University of Casablanca (UH2C), Casablanca, Morocco; 2 INSERM UMR-S-MD 1197, Ministère des Armées et Université Paris Saclay, Villejuif, France; 3 Laboratory of Cellular and Molecular Pathology, Faculty of Medicine and Pharmacy, UH2C, Casablanca, Morocco

**Keywords:** Leishmaniasis, Fc receptors, FcγRIIA, Immune response, ITAM

## Abstract

**Background:**

*Leishmania* is the causal parasite of leishmaniasis, a neglected tropical disease affecting millions of individuals worldwide, and its dissemination is linked to climate change. Despite the complexity and effectiveness of the immune response, the parasite has developed many strategies to evade it and take control of the host cell to replicate. These evasion strategies start at early stages of infection by hijacking immune receptors to mitigate the cellular response. In this study, we examined whether *Leishmania* uses the Fc receptor FcγRIIA/CD32a and its downstream signaling pathways to evade the host immune response.

**Methods:**

Regarding in vivo studies, CD32a transgenic mice and the corresponding wild types were infected with *Leishmania major* Friedlin strain. For the in vitro experiments, BMDMs isolated from WT or CD32a transgenic mice and control or CD32a knockdown differentiated THP-1s were infected with two species of *Leishmania*, *Leishmania major* and *L. tropica.*

**Results:**

In vivo, expression of FcγRIIA/CD32a was found to accelerate the signs of inflammation while simultaneously preventing the formation of necrotic lesions after *Leishmania* infection. In infected macrophages, the presence of FcγRIIA/CD32a did not affect the secretion of proinflammatory cytokines, while the balance between ITAMa and ITAMi proteins was disturbed with improved Fyn and Lyn activation. Unexpectedly, infection with *L. tropica* but not *L. major* triggered an intracytoplasmic processing of FcγRIIA/CD32a.

**Conclusions:**

Our observations underscore the significance of FcγRIIA/CD32a in cutaneous leishmaniasis and its potential use as a therapeutic target.

**Graphical Abstract:**

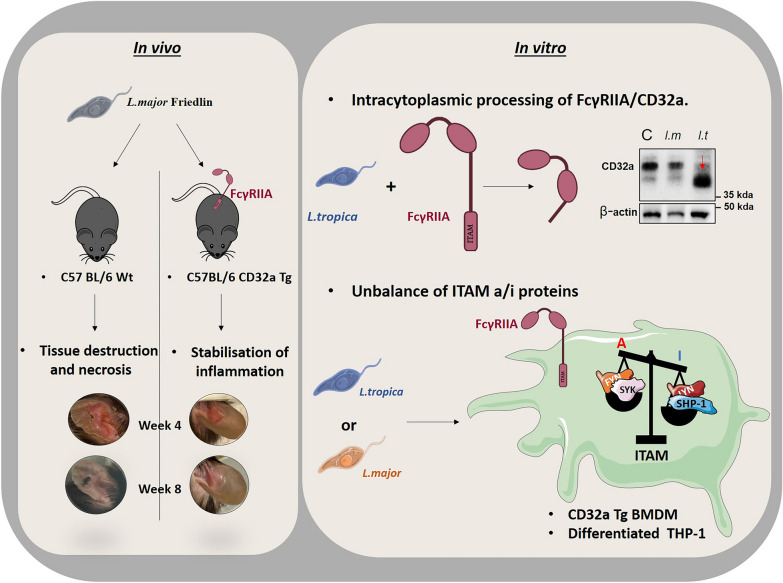

## Background

One of the most prevalent neglected tropical diseases worldwide is caused by the protozoa of the genus *Leishmania*. Three major forms of leishmaniasis exist, among which the cutaneous form affects an estimated 700,000 to 1.2 million individuals worldwide [1]. Despite the considerable incidence of this disease, the current treatments are limited because of their toxicity and treatment failures [2]. The alarming dissemination of leishmaniasis is attributed to multiple factors including climate and environmental changes, requiring major efforts to limit its detrimental impact [3]. During *Leishmania* infections, the parasite can modulate the host cell to orient several cellular processes towards its own survival and replication. Recent studies have shown how the *Leishmania* parasite can also utilize the host immune system to hijack its own surveillance [4–6]. Following a female sandfly’s bite, the promastigote form of the parasite is regurgitated into the host [7]. The parasite gets phagocyted by macrophages and is transported to the phagolysosome, where it transforms into the amastigote form. This form facilitates resistance to the hostile phagolysosome environment (acidic environment) and may spread to neighboring cells via phagocytosis of the infected cell without necessarily reaching its bursting point [8].

To initiate and give rise to an effective immune response against pathogens, the recognition of “threats” by specific receptors is critical. This function is ensured by pattern recognition receptors (PRRs) that identify specific molecular structures of the pathogens, also known as pathogen-associated molecular patterns (PAMPs) [9]. Therapeutically, proper understanding of the interactions between these receptors and their target molecules is core knowledge to develop efficient therapies. Immune cells can set up an effective early immune response if their immune receptors succeed in recognizing the danger at early stages. Among these receptors, Fc receptors are a family of glycoproteins that ensure various biological responses by which they can orient the immune response depending on the stimulus. By binding to the Fc portion of the IgG antibodies, Fc receptors initiate their immunological function. To ensure signal transduction upon receptor activation, Fc receptors possess specific motifs in their intracellular domain, namely, the immunoreceptor tyrosine-based activation motif (ITAM) and the immunoreceptor tyrosine-based inhibition motif (ITIM), which are critical for signaling pathway functioning [10].

The FcγRII receptor family includes glycoproteins inserted into the plasma membrane with conserved extracellular domains. FcγRIIA and FCγRIIC proteins have been reported to have similar cellular functions as activating-type Fc receptors while FcγRIIB was instead classified as functioning as inhibiting-type Fc receptors. Regarding FcγRIIA, studies have reported that the outcome of its activation depends on the stimulus [11]. In the context of infectious diseases, studies have discussed the potential involvement of Fc receptors and immunoglobulin in controlling *Plasmodium* infection while pointing out the challenges associated with using appropriate infectious models [12, 13]. Regarding *Leishmania* infection, the mechanism by which host macrophage surface receptors, such as Fc receptors, are modulated during leishmaniasis is poorly understood.

Our study aims to investigate the role of FcγRIIA receptor in the pathogenesis of cutaneous leishmaniasis in vivo as well as assess its involvement from the in vitro entry into the target cell to the involvement of ITAM signature pathway proteins after establishing the infection.

## Methods

### Culture, isolation of *Leishmania* and in vitro infection

Two species of *Leishmania, Leishmania major* and* L. tropica*, were used for the in vitro experiments. *Leishmania major* (MHOM/MA/17/Z04) was kindly provided by Dr. Lemrani Meryem (Leishmaniasis Laboratory at the Pasteur Institute of Morocco), and *L. tropica* (MHOM/MA/22/LC04) was isolated from skin lesions of Moroccan patients suspected of having a leishmaniasis skin infection at the Department of Dermatology (Ibn Rochd Hospital of Casablanca, Morocco). The serosites were aspirated by a doctor under sterile conditions using insulin-type intradermal syringes from the edge of the active skin lesion. These overall procedures were done according to the principles specified in the Declaration of Helsinki and in compliance with the local ethics guidelines (Biomedical Research Ethics Committee, Faculty of Medicine and Pharmacy, Hassan II University of Casablanca, Morocco; International Review Board 00002504). Both species were cultured in RPMI 1640 + GlutaMax (Gibco, USA) and 10% FBS (Biowest, France) and were used for in vitro experiments under ten passages to avoid loss of virulence.

*Leishmania major* Friedlin (*Lmj*F) was kindly donated by Dr. Pascale Pescher from the Pasteur Institute (Paris, France) and was cultured in M199 medium (Gibco) with l-glutamine and 10% FBS (Biowest, France) and then RPMI 1640 + GlutaMax (Gibco, USA) and 10% FBS (Biowest, USA); this strain was used for in vivo experiments under ten passages to avoid loss of virulence. Molecular identification and characterization of the *Leishmania* strains were performed by our team in Morocco at the Parasitology Laboratory of the Faculty of Medicine and Pharmacy (Hassan II University of Casablanca).

The in vitro infections of cells (5 · 10^5^ cells per well) were initiated by infecting cells with parasites at a ratio of 1:10.

To measure infectivity, *Leishmania major* and *L. tropica* parasites were stained with Hoechst Blue (Thermo Fisher Scientific, ref. H1399) at a final concentration of 1 ug/ml for 10 min, washed twice and added to the cells for 3-h infection. Following the incubation period, cells were washed to remove free promastigotes and then incubated for an additional 24 h, allowing the promastigotes to transform into amastigotes.

### In vivo experimental infection

CD32a transgenic animals were obtained from Dr. Ulrich Blank (Bichat Hospital, Paris, France) and were housed in our animal facility at Paul Brousse Hospital, Villejuif, France, where our in vivo experimentation was performed.

For the mouse models, the project received approval from local ethics committee no. 026 (ethics committee in animal experimentation) in France, Animal Use Protocol number APAFIS #39772-2022121310069256 v3. The experiments were performed in accordance with the guidelines of the French Council for Animal Protection and national ethics guidelines.

Wild-type C57BL/6 mice were purchased from Charles River to breed with CD32a transgenic mice. All mice were 6–8-week-old females with a C57BL/6 background.

Genotyping of mice was performed in our laboratory according to the protocol provided by Dr. Ulrich Blank. Briefly, DNA extraction from mouse tissues was performed. The primer sequences were: TgCD32a-F: CTG GTC AAG GTC ACA TTC TTC; TgCD32a-R: CAA TTT TGC TAT GGG C. The program was: 1, 5′ 94°; 20″ 94°; 30″ 64°; 35″ 72° (×12); 20″ 94°; 30″ 58°; 35″ 72° (× 25); 2′ 72°. Tissue digestion was done by adding 50 mM NaOH to the tissues and heating at 98° for 20 min. Next, 1 M Tris–HCl (pH 8) was added before centrifuging at 14,000 rpm for 10 min. Then, a PCR was done with the lysate (2 µl lysate/ reaction).

Mouse ear infection was initiated using a single intradermal inoculation (15 s/injection) at the earlobe in transgenic and wild-type mice using a single dose of parasite inoculum or 1 × PBS as a control. This injection site was not only chosen to mimic the natural course of the infection, since the mosquito typically targets humans' external limbs, but also to minimize the suffering of the animals as much as possible. The administered parasite dose was 10 µl at a concentration of 10^6^ parasites of parasite solution (inoculum) taken up in RPMI medium. This procedure was performed under brief general isoflurane gas anesthesia with an isoflurane flow rate per animal of 4% to 2% and an air flow rate of 0.4 l/min. This injection was executed at the beginning of the experiment and was the only injection. Weekly monitoring was carried out by measuring the diameter of the inflammation and lesions at the earlobe. Inflammation scoring (based on the average diameter of swellings and redness) was determined as previously described [14]. Daily monitoring was also carried out to observe the animal’s behavior. Twenty weeks post-injection, the mice were killed by cervical dislocation.

### Cell culture and reagents

THP-1 cells were cultured in RPMI 1640 + GlutaMax (Gibco) and 10% FBS (PAN biotechn). THP-1 cells were incubated with 50 ng/ml phorbol 12-myristate 13-acetate (PMA; sigma, UK) at 37 °C and 5% CO_2_ for 48 h to induce the differentiation of these monocytes into adherent macrophages. To inhibit the expression of the CD32a receptor, THP-1 cells were transduced with shRNA lentiviral particles (Santa Cruz Biotechnology, Inc.). THP-1 cells were plated and incubated with lentiviral particles for 48 h, and then cells were washed and replaced with fresh medium. Selection of transduced cells was performed by puromycin (3 µg/ml), and CD32a expression in resistant colonies was assessed by Western blotting.

Bone marrow-derived macrophages (BMDMs) were obtained from femurs of 6- to 12-week-old female C57/BL6 wild-type (Wt) (Charles River) and CD32 transgenic (CD32tg) mice. Bone marrow cells were thoroughly rinsed from the bone with Dulbecco's modified Eagle’s medium (DMEM + GlutaMAX, Gibco) with 10% FBS and 1% penicillin/streptomycin (Gibco) and cultured in 20% L929 cell culture medium to induce differentiation of bone marrow monocytes into macrophages. BMDMs were kept in culture at 37 °C, 5% CO_2_, for 5–7 days, after which they were harvested, counted and used.

### ELISA

The production of mouse IL-1ꞵ and mouse TNF-⍺ in the supernatant of infected cells was measured by ELISA (Enzyme-Linked ImmunoSorbent Assay) according to the manufacturer's instructions (R&D Systems, France).

### Western blot

Western blot analyses were performed with Bis–Tris gradient gels prepared in the laboratory using standard methods. Briefly, 5 · 10^5^ to 1 · 10^6^ cells/well were lysed on ice in TNT lysis buffer (50 mM Tris–HCL pH 7.4, 150 mM NaCl, 1% Triton X-100, 2 mM sodium pyrophosphate, 25 mM ꞵ-glycerophosphate, 1 mM sodium orthovanadate) supplemented with a protease inhibitor cocktail (Thermo Fisher Scientific), and the debris was removed by centrifugation at 10,000×*g* at 4 °C. Protein concentration was determined using a micro-BCA kit (Thermo Fisher Scientific). Samples were then boiled in SDS sample buffer (Novex) containing 10% ꞵ-mercaptoethanol (Sigma) and resolved on 5%–20% SDS–polyacrylamide gel electrophoresis. Immunoblot analysis was performed with specific antibodies, and antigen-antibody complexes were visualized by chemiluminescence (Immobilon Western, Merck Millipore) in the Bio-Rad ChemiDoc Imaging System.

### Antibodies

The antibodies used for this study were as follows: goat anti-FcγR/CD32 R&D systems, Cat#AF1875, 1/1000 dilution), rabbit anti-Syk (Cell Signaling, Cat#13198, 1/1000), rabbit anti-phospho-Syk (Cell Signaling, Cat#2710, 1/1000), rabbit anti-phospho-SHP-1 (Invitrogen, Cat#PA5-36682, 1/1000), rabbit anti-SHP-1 (Invitrogen, Cat#MA5-14839, 1/1000), rabbit anti-Fyn (Cell Signaling, Cat#4023, 1/1000), rabbit anti-Lyn (Cell Signaling, Cat#2796, 1/1000), rabbit phospho-Lyn (Cell Signaling, Cat#2731, 1/1000) and mouse anti-CD32 clone IV.3 (STEMCELL Cat# 60012, 1/1000).

### Flow cytometry

To analyze the expression of CD32a on the cell surfaces, differentiated THP-1 cells or BMDMs were infected with the two species of *Leishmania* for 30 min, 1 h, 2 h and 3 h and then washed three times before being detached with accutase. Cells then were incubated with mouse anti-CD32 clone IV.3 for 30 min in the dark on ice. After washing, cells were incubated with Alexa Fluor® 488 AffiniPure™ Donkey Anti-Mouse IgG for 30 min in the dark on ice and then washed and suspended immediately in 250 ml cold RPMI 1640 and analyzed immediately.

The degree of infectivity was assessed after infecting differentiated THP-1 cells and BMDMs with Hoechst-stained *L. major* or *L. tropica* for 3 h, washed three times and then incubated for 24 h at 37 °C. Next, the cells were washed three times before being detached with accutase and then suspended immediately in 250 ml cold RPMI 1640 for analysis.

For phagocytosis assay, fluorescent beads [FluoSpheres™ carboxylate-modified microspheres, yellow-green fluorescent (505/515), ThermoFisher] were incubated with BMDMs and differentiated THP-1 for 2 h. The cells were washed three times before being detached with accutase and suspended immediately in 250 ml cold RPMI 1640 for analysis.

Cells were analyzed by Fortessa flow cytometer (Becton Dickinson). Results were analyzed using Flowjo software X (Ashland, OR, USA). Results were represented by median fluorescence intensity (MFI).

### Statistical analyses

All data were obtained using cells from at least three independent culture preparations or at least three independent animals per genotype. Statistical analysis was performed using GraphPad Prism 8.4 software by applying unpaired t-test and one-way ANOVA tests. *P* values < 0.05 were considered statistically significant (GraphPad Software, Inc., La Jolla, CA, USA).

## Results

### Assessment of localized lesion development in wild type mice compared to transgenic mice expressing CD32a

**Fig. 1:**
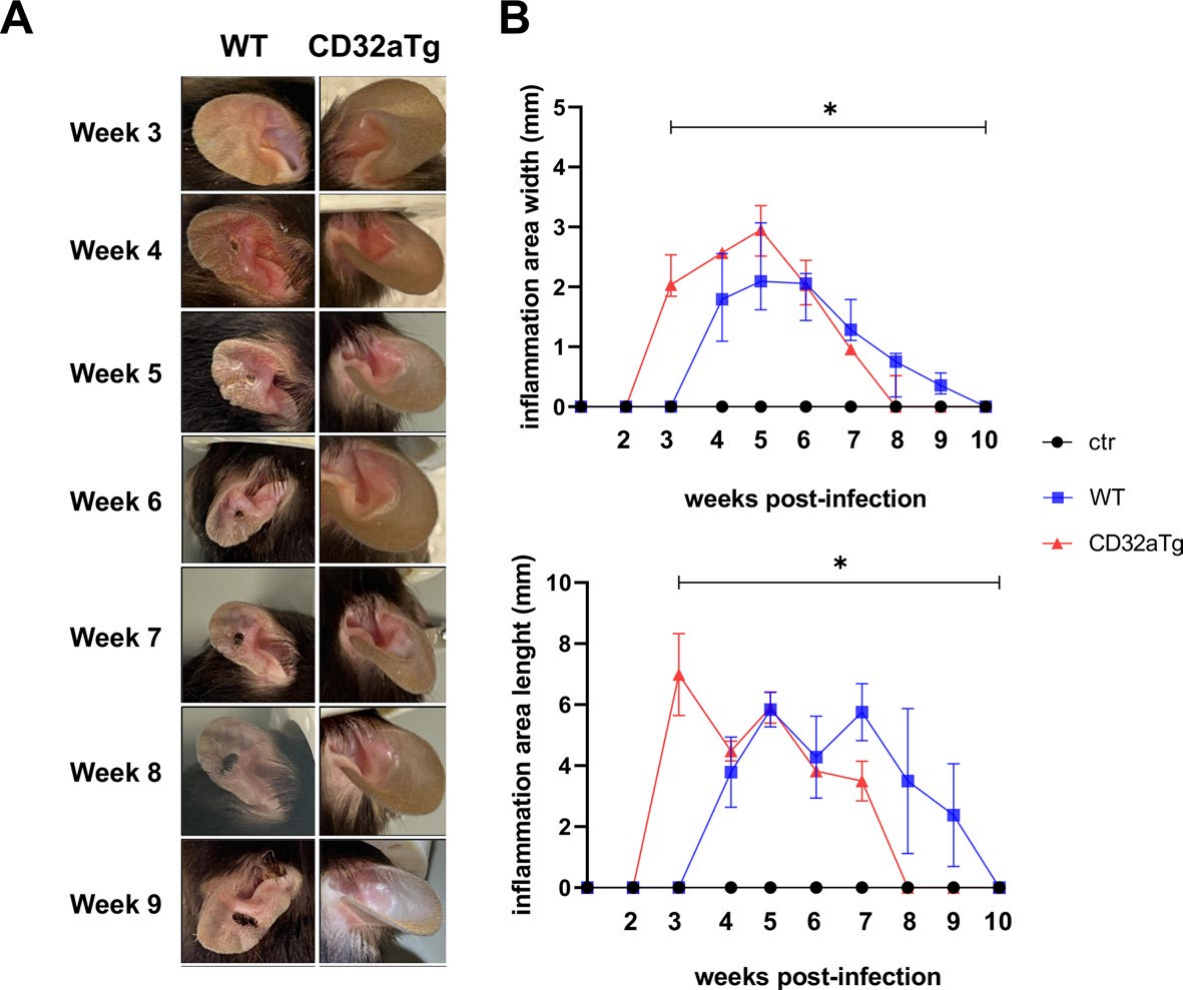
Comparison of localized lesions between wild-type and CD32a Tg mice. *Leishmania major* Freidlin strain was injected into the ear of C57BL/6 Wt or CD32a Tg mice; then, the lesions were observed starting from 3 to 9 weeks in each genotype. Images of the evolution of lesions through time are shown (**A**). In parallel, the length and width of the inflammation area (swelling and redness) were measured (**B**). The results are represented as median with interquartile range; number of mice per groups = 5; number of experiments = 2. Statistical significance is indicated by *P < 0.05 compared to the control group (PBS injected)

*Leishmania major* is known to trigger inflammation and cutaneous lesions. Hence, to assess the impact of the Fc receptor FcγRIIA/CD32a on the inflammation induced by *Leishmania* infection in vivo, transgenic mice expressing CD32a (CD32a Tg) and the corresponding wild-type (WT) genotype mice were infected with the *L. major* Friedlin strain parasite. The choice of CD32a could be justified by the fact that CD32a is not expressed in mouse [11], making the CD32a Tg mouse a suitable model to explore the role of this Fc receptor in *Leishmania* pathophysiology. The parasite was then injected into the ears of CD32a Tg and WT mice. Four weeks post-infection (p.i), the development of a necrotic lesion was observed in WT mice, and the lesion was surrounded by swelling and redness as markers of inflammation. The initial lesion increased in size throughout the weeks, stabilizing at 8 weeks p.i. (Fig. 1A). A scabrous texture around the edges of the ear, which might have been a sign of dehydration, was also noticed in most WT mice at the end of the experiment (Fig. 1A). In contrast, at 4 weeks p.i., inflammation but no tissue destruction was detected in the ears of CD32a Tg mice, suggesting that the presence of FcγRIIA/CD32a prevents tissue necrosis induced by the *Leishmania* infection (Fig. 1A).

After injecting the parasite into the ears, the development and progression of the inflammation were monitored by measuring the width and length of the swelling/redness with a sliding caliper for 10 weeks. Two weeks after injection, inflammation was observed in the CD32a transgenic mice but not in the WT mice, reaching a peak within 3 weeks for the lengths and 5 weeks for the width; it finally was resorbed at 8 weeks p.i (Fig. 1B). In contrast, the development of inflammation in WT mice appeared later, around 4 weeks p.i, coinciding with formation of the lesion, and finally disappeared within 10 weeks. It appears therefore that there is a significant difference in the development of inflammation between WT and CD32a Tg mice (Fig. 1B). Hence, inflammation seems to take place earlier in CD32a Tg mice than in the WT, which may explain the prevention of tissue necrosis after infection.

### Expression of the FcRγIIa/CD32a after *Leishmania* infection

**Fig. 2:**
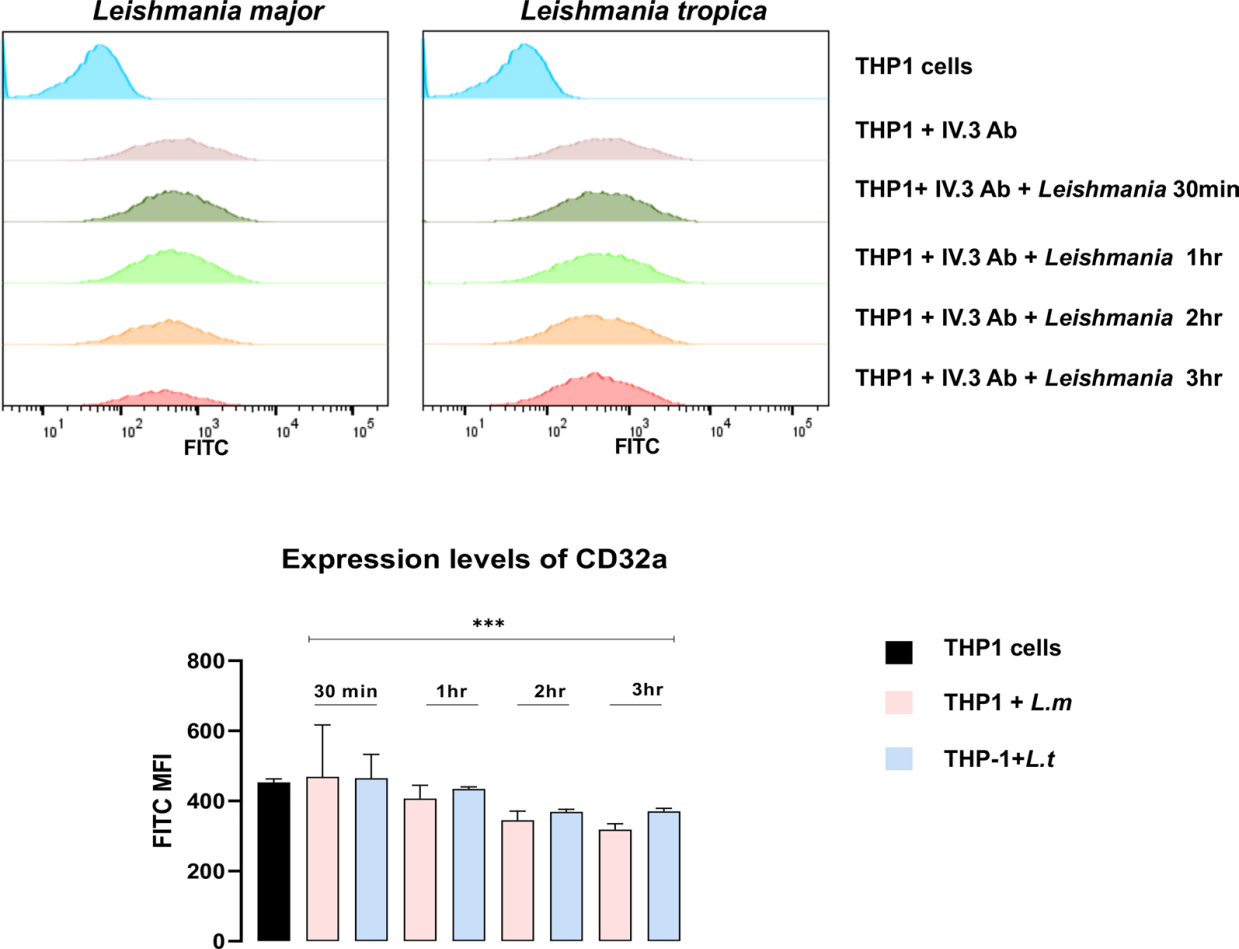
Expression of FcγRIIA/CD32a after infection with *Leishmania*. Differentiated THP-1 cells were infected for 30 min, 1 h, 2 h and 3 h with *Leishmania major* or *L. tropica*; next, CD32a expression was assessed by flow cytometry. The results are represented as median with interquartile range; number of experiments = 3. Statistical significance is indicated by ***P < 0.001 compared to the control non-infected group. MFI: mean fluorescence intensity

Since our in vivo observations suggest a role of FcγRIIA/CD32a in the control of pathology caused by *Leishmania*, the impact of the parasite on the expression level of the immunoreceptor was investigated. After infecting THP-1 cells with *L. major* or *L. tropica* promastigotes, a gradual slight decrease in the cell surface expression of CD32a in both species was observed by flow cytometry (Fig. 2). Moreover, no significant differences were noticed between *L. major* and *L. tropica* in terms of their ability to decrease cell surface expression of CD32a (Fig. 2).

**Fig. 3:**
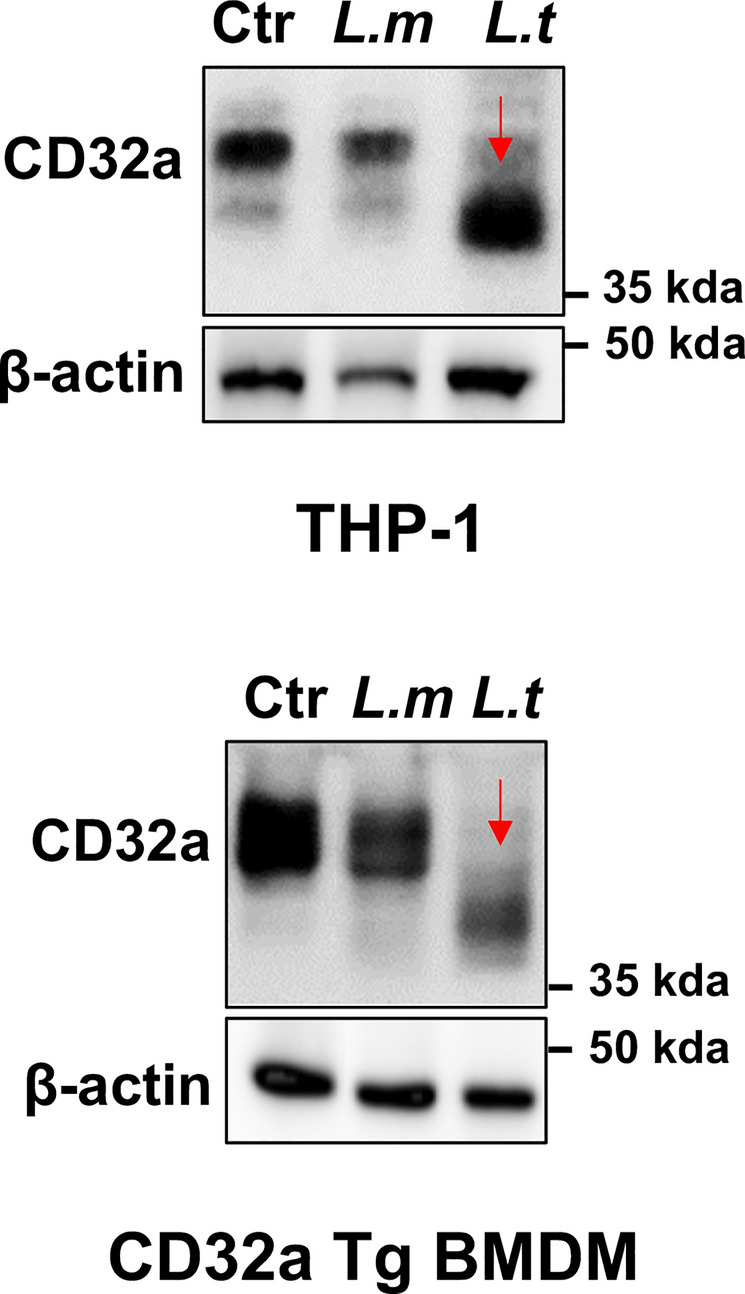
Profile of CD32a expression in THP-1 cells and CD32a Tg BMDMs after *Leishmania* infection. Differentiated THP-1 cells or CD32a Tg BMDMs were infected for 3 h by *Leishmania major* (*L.m*) or *L. tropica* (*L.t*). CD32a was then analyzed by Western blotting in cell lysates compared with uninfected cells (Ctr) (number of experiments = 3). β-Actin was used as a loading control. The red arrow indicates the processed form of CD32a

The expression panel of the FcγRIIA/CD32a was also assessed by Western blotting (WB) in THP-1 cells as well as in BMDMs isolated from CD32a Tg mice 3 h after infection with both species of *Leishmania* (Fig. 3). While the WB confirmed the small decrease in FcRyIIa/CD32a expression following *L. major* infection, surprisingly, following *L. tropica* infection, CD32a appeared as a shorter form in both cell types (Fig. 3). Since both species of *Leishmania* induced a slight but significant reduction in the cell surface expression of CD32a (Fig. 2), the shorter isoform observed in WB after infection with *L. tropica* is likely the result of a cleavage of CD32a in its intra-cytosolic domain. Such a difference in the expression profile of FcγRIIA /CD32a in WB after infection with both species of *Leishmania* was unexpected.

**Fig. 4:**
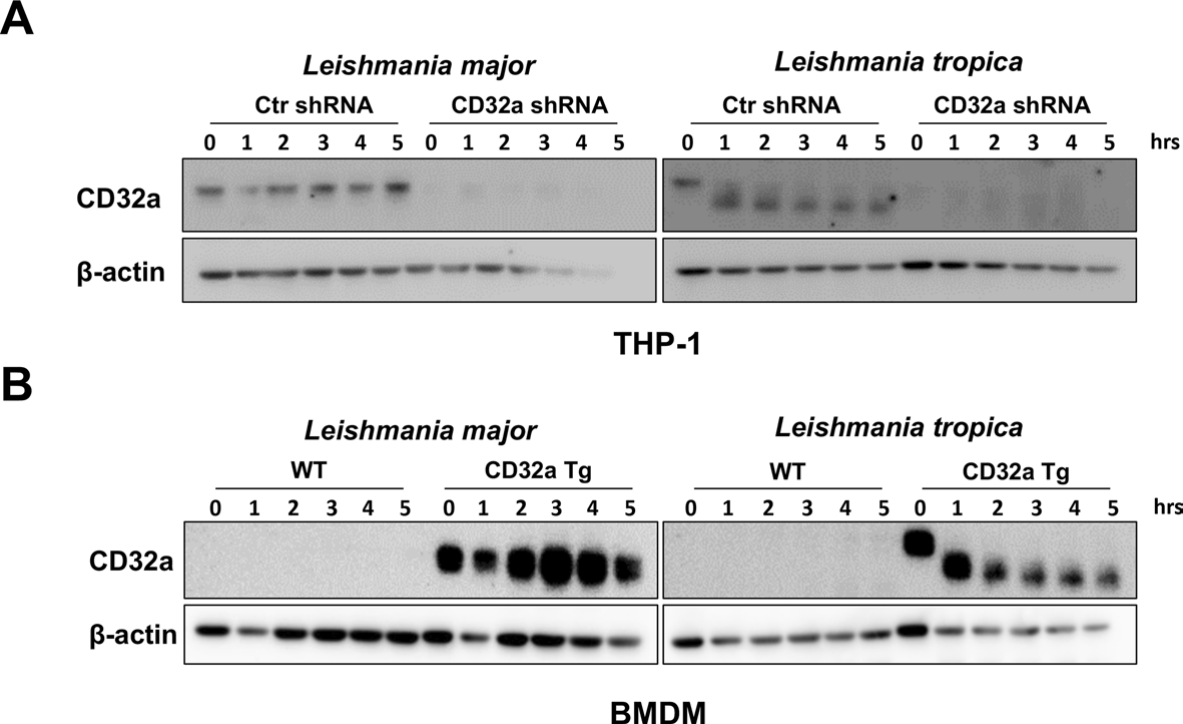
Only *Leishmania tropica* infection promotes the processing of CD32a. Differentiated control scramble shRNA (scr) and CD32a shRNA knockdown THP-1 cells (**A**) or WT and CD32a Tg BMDMs (**B**) were infected for the indicated times by *Leishmania major* or *L. tropica*. CD32a was then analyzed by Western blotting in cell lysates (number of experiments = 3). β-Actin was used as a loading control

To further explore the unexpected processing of FcγRIIA/CD32a, a kinetic study of infection was performed using THP-1 cells. We noticed that the cleavage of CD32a appears soon after 1 h post-*L. tropica* infection and even earlier, i.e. 15 min (data not shown), while *L*. *major* infection did not induce a cleavage even after 5 h (Fig. 4A). Similar results were observed when infecting BMDMs isolated from CD32 Tg mice with both species of the parasite (Fig. 4B). The use of CD32a-deficient THP-1 cells (knockdown performed with shRNA) and the absence of CD32a in WT BMDMs confirmed the specificity of the anti-CD32a used in WB (Fig. 4). Finally, cell extracts from THP-1 were also incubated with cell extracts from *L. tropica* promastigotes but the co-incubation did not promote the specific processing of CD32a, suggesting that a protease of the protozoan is not involved in this process (data not shown).

### Role of FcγRIIA/CD32a in *Leishmania* entry and phagocytosis

**Fig. 5:**
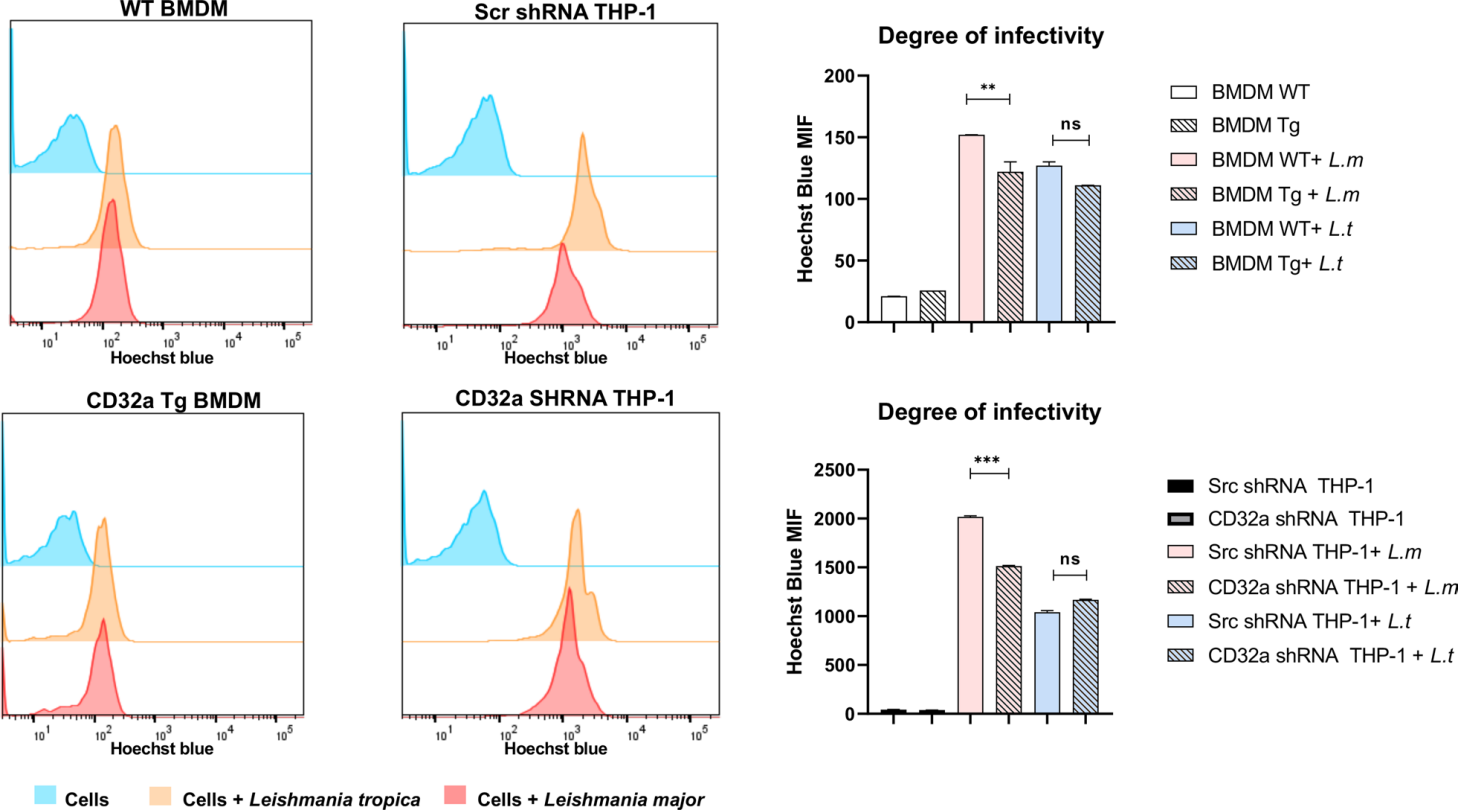
Impact of FcγRIIA/CD32a on *Leishmania* infectivity. WT and CD32a Tg BMDMs or differentiated control scramble shRNA (scr) and CD32a shRNA knockdown THP-1 cells were infected for 3 h with Hoescht-fluorescent *Leishmania major (L.m)* or *L. tropica (L.t)* and next washed and incubated for further 24 h. The degree of infection by the parasite was then assessed by flow cytometry by measuring the fluorescence inside the cells. The results are represented as median with interquartile range; number of experiments = 3. Statistical significance is indicated by ***P < 0.0005, **P = 0.0095 when cells (WT BMDMs and Tg BMDMs or THP-1 and shRNA CD32a knockdown THP-1) infected with the same parasite species were compared. MFI: mean fluorescence intensity

**Fig. 6:**
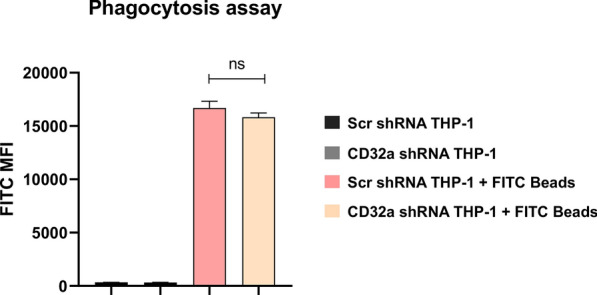
FcγRIIA/CD32a does not affect the phagocytosis function of macrophages. Differentiated control scramble shRNA (Scr) and CD32a shRNA knockdown THP-1 cells were incubated for 2 h with fluorescent beads and then washed thoroughly to be analyzed using flow cytometry to measure the degree of fluorescence inside the cells. The results are represented as median with interquartile range (number of experiments = 3). *MFI* mean fluorescence intensity

As an immunoreceptor localized at the surface of monocytes/macrophages, we investigated whether FcγRIIA/CD32a affects the entry of *Leishmania* into its target cell, which occurs through phagocytosis [15]. WT or CD32a Tg BMDMs as well as differentiated THP-1 or CD32a-deficient THP-1 cells were infected with both *Leishmania* species to measure the degree of infectivity using flow cytometry (Fig. 5). Whereas FcγRIIA/CD32a did not seem to affect the infectivity of cells by *L. tropica*, divergent results were obtained for *L. major*. Indeed, in BMDMs, the expression of CD32a decreased the infectivity, whereas in THP-1 the knockdown of this immunoreceptor resulted in reduced infectivity (Fig. 5). We currently have no explanations for this discrepancy. However, this observation regarding *L. major* may only be an epiphenomenon since the knockdown of CD32a did not affect the phagocytotic ability of the differentiated THP-1 as assessed using fluorescent beads (Fig. 6).

### FcγRIIA/CD32a promotes improved Lyn activation but decreased SHP-1 activation after *Leishmania* infection

Like the other Fc receptors, FcγRIIA/CD32a possesses an immunoreceptor tyrosine-based activation motif (ITAM) in its intracellular domain that is important for signal transduction pathways once the receptor is activated [10]. Hence, activation of FcγRIIA/CD32a triggers the recruitment of Fyn, a src family protein tyrosine kinase (PTK), which phosphorylates the ITAM motif on two tyrosine residues, resulting in the protein recruitment of the PTK Syk family and transmission of an activator signal [10].

**Fig. 7:**
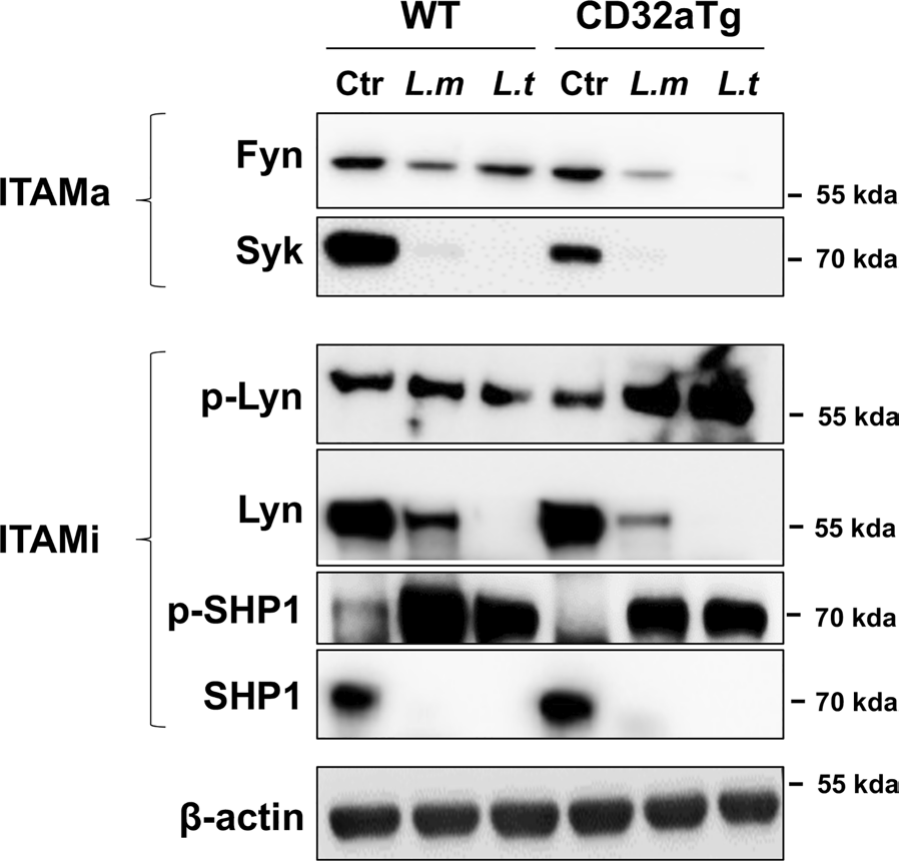
ITAMa and ITAMi signature in WT or CD32a Tg BMDMs after *Leishmania* infection. WT and CD32a Tg BMDMs were infected for 3 h with *Leishmania major* or *L. tropica*. Then, cell lysates were analyzed by Western blotting for the indicated proteins corresponding to ITAMa or ITAMi signature compared with uninfected cells (Ctr) (number of experiments = 3). β-Actin was used as a loading control

WT and CD32a Tg BMDMs were infected with both *Leishmania* species; then, Fyn and Syk were analyzed by WB. Surprisingly, in both genotypes, infection with protozoa triggers the disappearance of Syk (Fig. 7). Likewise, Fyn was less detectable in the cell extracts following infection, with a significant reduction observed when the cells expressed CD32a in addition to the other Fc receptors present on the BMDM surfaces (Fig. 7). Hypothetically, we speculate that both kinases were either degraded or moved into the insoluble fractions, rendering them undetectable in WB, which we believe is more plausible.

After years of the ITAM motif being characterized as activated in the cellular response, it appears that the ITAM can also induce inhibitory responses. This signal is known as ITAMi [16]. Under certain conditions, one of the two tyrosine residues within the ITAM is phosphorylated by the Src kinase Lyn. With two juxtaposed receptors presenting mono-phosphorylated ITAMs, the phosphatase SHP-1 is recruited and then phosphorylated on Tyr536 by Lyn. Consequently, this phosphorylation leads to an inhibition of cell activation [11]. According to our results, we observed that *Leishmania* infection also triggers the loss of Lyn and SHP-1; however, only in BMDMs expressing CD32a was a significant activation of Lyn detected, as assessed by its phosphorylated form (Fig. 7). Furthermore, SHP-1 appeared to be more phosphorylated and thus activated in the WT BMDMs compared to the CD32a Tg BMDMs (Fig. 7).

### FcγRIIA/CD32a does not affect the production of proinflammatory cytokines

**Fig. 8:**
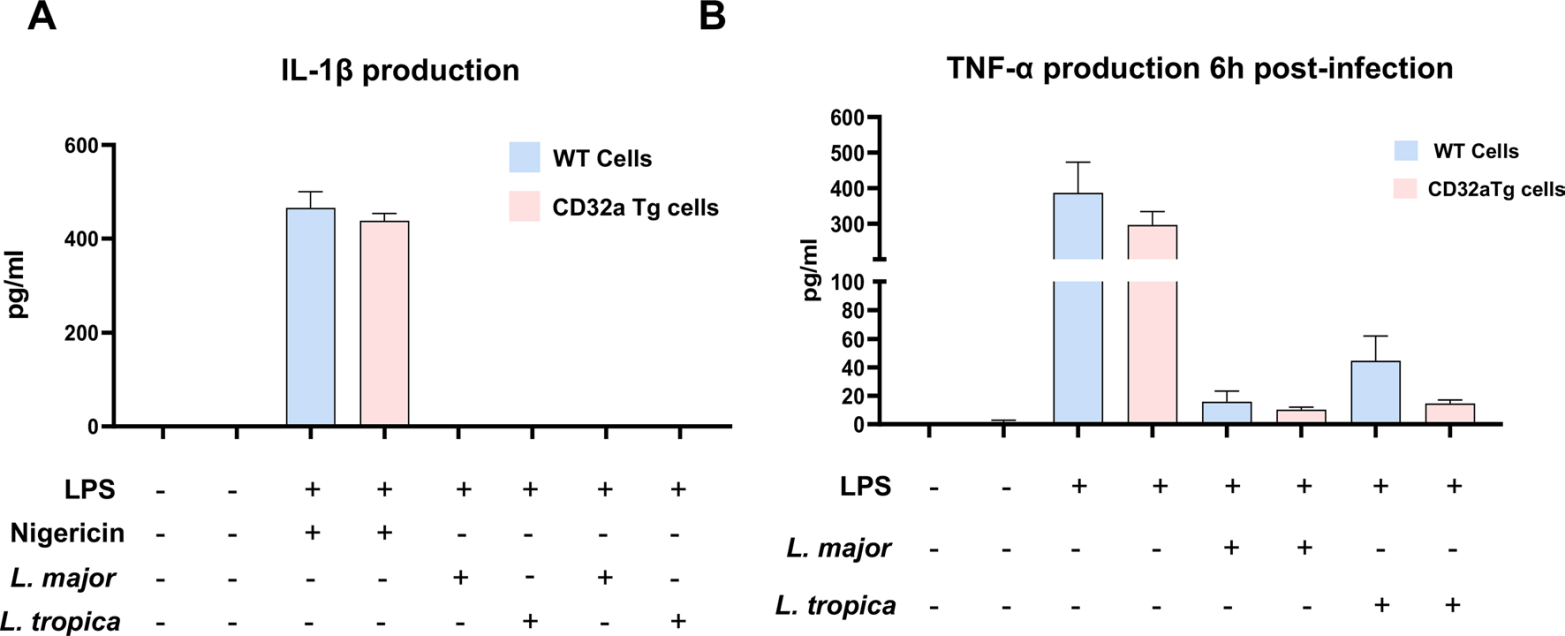
The production of proinflammatory cytokines is not affected by FcγRIIA/CD32a. **A** WT BMDMs or CD32a Tg BMDMs were primed with LPS (100 ng/ml for 2 h) and then infected for 6 h by *L. major* or *L. tropica*. As a positive control, after LPS priming, cells were incubated with nigericin (10 mM) for 45 min. The supernatants were collected and analyzed by ELISA for the presence of IL-1ꞵ. **B** WT BMDMs or CD32a Tg BMDMs were infected for 6 h by *L. major* or *L. tropica* or treated with LPS (100 ng/ml). The supernatants were next collected and analyzed by ELISA for the presence of TNF-α. The results are pooled and are represented as median with interquartile range (number of experiments = 3)

Activated/phosphorylated Lyn, upon infection in BMDMs expressing CD32a, has been reported to directly activate Syk, leading to a stimulation of MAPK and NF-kB signaling pathways [17]. To assess a potential involvement of the FcγRIIA/CD32a in the inflammatory response, the production of proinflammatory cytokines in *Leishmania*-infected WT or CD32a Tg BMDMs was investigated. Infection of BMDMs with *Leishmania* has been reported to trigger NLRP3 inflammasome activation, leading to the release of mature IL-1b [18]. Nonetheless, infection of BMDMs with either *L. major* or *L. tropica*, even after LPS priming, did not lead to the secretion of IL-1b (Fig. 8A). Similar observation was noted upon 24 h infection (not shown). As a positive control, priming with LPS followed by nigericin treatment induced the production of IL-1b, which was not affected by the expression of CD32a (Fig. 8A).

Likewise, and although *Leishmania* infection of peritoneal inflammatory macrophages results in the production of TNFα [19], in our model, infection with both species of *Leishmania* did not promote significant TNFα production (Fig. 8B). In contrast, activation of TLR4 with LPS induced TNFα production. Notably, the presence of CD32a did not alter the levels of this proinflammatory cytokine. Surprisingly, TNFα production was significantly inhibited after TLR4 activation in *Leishmania*-infected BMDMs (Fig. 8B).

## Discussion

*Leishmania* parasites have developed several ways to evade the immune system to colonize their hosts. Among these, *Leishmania* alters intracellular signaling pathways, which facilitates the survival and replication of the parasite [20]. Here, we investigated the impact of the Fc receptor FcγRIIA/CD32a on cutaneous leishmaniasis. In our mouse model, we used primary strains of *Leishmania major* or *L. tropica* isolated from Moroccan patients but none triggered lesions once injected into the ears of mice, demonstrating therefore the importance of the species barrier and the involvement of host factors in the pathogenesis of cutaneous leishmaniasis. We then used the *L. major* Friedlin strain, a reference strain, to infect WT or CD32a Tg mice. Interestingly, expression of CD32a prevented the formation of necrotic lesions in the ear a few days after injection of the parasite, while in those animals, signs of inflammation (swelling and redness) appeared earlier. This rapid occurrence of inflammation in earlier weeks of *Leishmania* infection may prevent the damaging effect of the protozoan. Unlike humans, mice do not express CD32a. Nevertheless, the observation that Tg mice expressing this Fc receptor seem to be protected from the detrimental effects of *Leishmania* infection shows that FcγRIIA/CD32a is a promising target for curing cutaneous leishmaniasis.

Regarding Fc receptor engagement, it is well known that these receptors typically bind to immunoglobulins, leading to an immune response. However, we chose not to opsonize the promastigotes with infected mouse serum prior to in vitro infection. This decision was supported by evidence that certain microorganisms, such as *Escherichia coli* [21], *Candida albicans* [22] and certain molecules like β-glucan (widely present on microorganisms) [23], can activate ITAM pathways independently of immunoglobulins, suggesting that receptor signaling can occur through alternative mechanisms. For *Leishmania*, it has been observed that the parasite secretes soluble components capable of engaging ITAM-bearing receptors directly [5], possibly bypassing the need for antibody-mediated opsonization. Although opsonizing promastigotes or using lesion-isolated amastigotes could enhance receptor signaling, our approach allowed us to investigate whether FcγRIIA/CD32a activation might occur independently of immunoglobulin binding. This choice does not preclude opsonization’s potential to amplify receptor engagement, as shown for CD32a signaling in other contexts [11], which may enhance the immune response and should be considered in future studies.

The mechanisms by which FcγRIIA/CD32a may accelerate inflammation after *Leishmania* infection remain unclear. Although this Fc receptor did not affect the secretion of proinflammatory cytokines, it is associated with ROS production in neutrophils during parasitic infections like malaria [24]. ROS production has been linked to neutrophil apoptosis and cell death, which prevents tissue pathology [25]. This Fc receptor does not seem to significantly modify the infectivity of macrophages by *Leishmania* or phagocytosis. Investing longer incubation periods (48 to 72 h) may provide further insight into potential differences in parasitic growth. At the molecular level, expression of FcγRIIA/CD32a surprisingly improves activation of the Lyn kinase, which is known to play a role as a break in the activation of several cell types [26, 27]. Lyn has been reported, among other Src family kinases, to play a key role in the uptake of *Leishmania* amastigotes by macrophages and inhibition of these proteins reduces disease severity [28]; hence, Lyn activation in CD32a Tg cells may stimulate phagocytosis of parasites, accelerating innate immune response and thus inflammation.

Lyn suppresses ITAM signaling through the phosphorylation of SHP-1 [29]. Curiously, FcγRIIA/CD32a appears to decrease SHP-1 activation after *Leishmania* infection. *Leishmania*-induced macrophage SHP-1 activity is necessary for its survival within phagocytes because of attenuation of nitric oxide-dependent and -independent microbicidal mechanisms [30] so that the *Leishmania* replication can be mitigated in the context of FcγRIIA/CD32a expression, which may explain the lack of lesions. SHP-1, a member of the protein tyrosine phosphatase (PTP) family, is essential for inhibitory signaling pathways in hematopoietic cells [31]. Loss of SHP-1 protein leads to increased mortality in mice due to exacerbated inflammation and difficulties in recruiting macrophages and neutrophils to wound sites during infection [32]. *Leishmania* exploits SHP-1 to evade the immune response by releasing a ligand that binds to a receptor coupled with an Fc receptor chain, Mincle, shifting the cellular response toward inhibition. In Mincle-deficient cells, stronger activation of dendritic cells and a Th1 response is observed, correlating with reduced parasite load [5].

Our results suggest that expression of FcγRIIA/CD32a tends to trigger an ITAMi signature based on the observation of p-Lyn and p-SHP-1, the active form of both proteins. After infection, Lyn and SHP-1 disappeared from the cell extract but they are probably not degraded since their phosphorylated/activated forms are still detectable. Rather, Lyn and SHP-1 likely move into a non-soluble fraction after their activation, as described for some adaptors in innate immunity [33]. Similarly, Fyn is probably only activated in CD32a Tg phagocytes after infection, representing an ITAMa signature. Thus far, Fyn has not been reported to have a direct link to leishmaniasis, but it has been shown that inhibiting Fyn/Lck kinase activity prevents Treg differentiation into Th17 cells through the AKT/mTOR pathway [34]. Th17 cells play a controversial role in cutaneous leishmaniasis as some reports suggest that they confer resistance against different species of *Leishmania* (in both visceral and cutaneous leishmaniasis) [35]. Notably, during leishmaniasis, IL-17, the main cytokine released by Th17, plays a crucial role in cutaneous leishmaniasis by promoting the migration of neutrophils and macrophages to infection sites and promoting macrophage differentiation into an M2 phenotype, favoring a good environment for *Leishmania* invasion [36].

Previous studies have shown that *Leishmania* parasites can induce the production and secretion of various immunosuppressive molecules, which then inhibit the production of proinflammatory cytokines in macrophages [20], allowing them to remain hidden and replicate safely inside the cell. We have investigated whether FcγRIIA/CD32a expression would change this outcome. In our hands, infection of BMDMs with *Leishmania* did not lead to the production of proinflammatory cytokines such as IL-1ꞵ and TNF-α. While Lyn suppresses ITAM signaling through the phosphorylation of SHP-1, which regulates TLR pathways that trigger inflammatory responses to pathogens [29], FcγRIIA/CD32a did not affect the secretion of TNFa after TLR4 stimulation or the production of IL-1b after NLRP3 inflammasome activation.

Finally, infection of cells expressing CD32a by *L. tropica*, but not *L. major*, led to the cleavage of the receptor, resulting in a loss of approximately 10 kDa and a weight of around 30 kDa. To date, we are the first to our knowledge to report such cleavage in the context of *Leishmania* infection. It has been previously shown that activation of FcγRIIA/CD32a in platelets through crosslinking with the protein GPVI results in the cleavage of the receptor by calpain [37]. However, attempts to inhibit the cleavage of CD32a in our model with calpain or other protease inhibitors were unsuccessful (data not shown), requiring further studies to understand the molecular mechanism and function of this processing. We hypothesize that this processing in the intracytoplasmic domain of CD32a may favor downstream signaling, as suggested by the observation of increased Fyn and Lyn activation upon *L. tropica* infection (Fig. 7).

## Conclusions

Our study proposed a mechanism by which *Leishmania* may dampen the host immune system, precisely by altering intracellular signaling pathways to inhibit activation of the cellular response. Further studies are however required to fully understand the link between FcγRIIA/CD32a and inflammation in the context of *Leishmania* infection.

## Data Availability

No datasets were generated or analysed during the current study.
